# Opinion—Bristol's Fourth Major Hospital

**Published:** 1973-07

**Authors:** James C. Briggs

**Affiliations:** Consultant Pathologist, Frenchay Hospital, Bristol, Honorary Secretary, Bristol Civic Society


### Opinion

### BRISTOL'S FOURTH MAJOR HOSPITAL

Traditional hospital medical care centres on activity within the hospital itself; very little consideration is given to the effect of a hospital's presence on its neighbourhood, on persons other than patients, or on lines of public transportation to and from its precinct. With this type of care the evolution of an individual hospital is based almost entirely on internal considerations: planning stops at the boundary fence. Evolution of total hospital care in a town like Bristol is equally based on parochial factors. People certainly look at facts and figures, population studies, etc., when making a decision about, say, how many hospitals Bristol needs, but this, I suggest, is not enough.

I sat on a Regional Hospital Board/United Bristol Hospitals/University Committee back in the late 1960s. This was convened to advise about the future shape of Bristol's hospital and medical services. It was the second attempt in a few years to produce such a report. The first had been dominated by Departmental (then Ministerial) norms—so many geriatric beds per 100,000 population, and so on.

There had never really been a need to call the first Committee, since its answer was automatic and entirely dictated by mathematics. The Chairman of the second committee said that such an exercise was not to be undertaken again — Departmental norms could and would not be the over-riding factors. This was a joy to hear and we talked about the need to consider not only the patient, but his relatives as well. "Keep the patient near his home" was one of our mottoes. We came up with a report which, at the time, I was proud to be associated with. It was in advance of its time, but, in retrospect, I now realise was contaminated by traditional hospital planning limitations. The report was subsequently replaced by the third and current attempt to look at the problem. I was not involved in this but I suspect that it was conceived within the traditional brief.

Why am I critical of the traditional brief? Simply because it is too narrow—it fails to consider factors which should be considered and uses only established questions and answers to reach a conclusion. (It is by no means unique in this matter.)

The sort of question traditional hospital planning asks is, "how much will it cost?", "what sort of services shall we put inside the hospital?". It very rarely asks "is there a **social** (as distinct from medical) need?". I certainly never asked myself this question in the late 1960s when I sat on that committee and we discussed the need for a District General Hospital in South Bristol. If I had done, then the answer "yes" would have screamed at me. As it was the question never really arose and it was only later, after I had become Secretary of the Bristol Civic Society, that I really became aware of some of the problems that face people who live in South Bristol.

There are further questions that the planners of Bristol's future hospitals ought to ask, but, before laying them out it is not amiss to outline broadly the questions which are routinely asked about most developments in this commercial age. Before a project is accepted (and assuming someone has made a case for it in the first place) then someone, somewhere says a) Is it politically acceptable?

b) Is it technically feasible?

c) Will the returns be satisfactory?

If the answer to these questions is "yes" then the finance is found at some stage (often dictated by question a).

There are three other questions which ought to be asked, and very rarely are; they are:—

a) What will be the effect of this project on the individual?

b) What will be the effect of this project on Society?

c) What will be the effect of this project on the Environment?

Sometimes these questions produce negative answers, whereas the first three may have been affirmative. A good many people think that the Aswam High Dam would not have been built in its present form if the Environment question had been posed beforehand.

Are these last questions really valid when one thinks of Bristol's hospital needs? Not only do I think "yes" but I think they are vital.

a) The individual. Hospitals are to serve patients and not the persons who work in them. Any new hospital should therefore be sited not near the nearest motorway junction where the peripatetic doctor can most easily reach it, but as near as possible to the centre of population that the hospital intends to serve. To plan a hospital based on other than this consideration automatically implies that the patient has to travel further than necessary: he must use public transport, or his own car. Those of you, if any, who have tried to reach Frenchay or Southmead hospital by bus from South Bristol must know how time consuming and exhausting this can be. To believe that all patients will have their own transport shows supreme ignorance of the energy crisis which the world seems to be heading for.

b) Society. As far as hospitals are concerned Society equals patients and their relatives. It is evident that a new South Bristol Hospital at Whitchurch Airport would be far more accessible to the great majority of the hospital's "society" than one, for instance, at Barrow Gurney. To ignore this as a consideration could result in a planning disaster. Besides this, a local population often becomes greatly attached to a hospital in its midst. One only has to think of the affection given by local inhabitants to Cossham Hospital to see this illustrated. Such ties could well be forthcoming around Whitchurch. There is virtually no indigenous population around Barrow Gurney.

c) Environment. Hospitals generate traffic. New hospitals should be constructed, where possible, near existing and well used lines of public transport. Bus companies are reluctant to run services for social causes and the National Bus Company, which owns the majority of Bristol's buses, must, by law, make a profit. There are no city bus services between North East and North West Bristol that do not go through the City Centre; the company does not run arcuate routes between the areas served by Frenchay Hospital and Southmead Hospital because it doesn't pay. The chances of an adequate service to Barrow Gurney are only slightly better. At the moment Barrow Gurney Hospital is served by three country bus routes, all starting from the Central Bus Station and running along the A38. This radial route could possibly be improved and still remain within the National Bus Company's brief. The chances of decent connecting arcuate services are, in my opinion, just as remote as those in North Bristol. From South Bristol Barrow Gurney would be more difficult to reach than the United Bristol Hospitals. Patients would still largely use these latter hospitals, thereby partly defeating the object of the fourth hospital.

There remain two further potential sites owned by the Department of Health. One of these—Winford Hospital, falls into virtually the same category as Barrow Gurney and consequently I don't think it would prove to be adequate.

The last potential site is Brislington House, situated on the A4 to Bath just inside the City boundary. Several country buses run past the door and a number of City buses serve the area, including a rare example of an arcuate service (from Withywood, Hartcliffe, Knowle West and Knowle). This route also runs near the Whitchurch site and, as a result, both sites are well served by existing bus services.

The three "second tier" questions posed earlier all produce the same sort of answer. The sites at Barrow Gurney and Winford do not meet requirements. There are only two practical sites (short of paying vast sums to purchase fresh land); they are Whitchurch Airport and Brislington. Having established this then I think it is up to the Planners to make a choice between these two using traditional criteria. I wonder if they will agree with my arguments?

**James C. Briggs, M.B., B.S., D.Path., M.R.C.Path., Consultant Pathologist, Frenchay Hospital, Bristol, Honorary Secretary, Bristol Civic Society.**

